# Bis{4-bromo-2-[(naphthalen-1-yl­imino)­meth­yl]phenolato-κ^2^ *N*,*O*}copper(II)

**DOI:** 10.1107/S1600536812004989

**Published:** 2012-02-10

**Authors:** Gholam Hossein Shahverdizadeh, Seik Weng Ng, Edward R. T. Tiekink, Babak Mirtamizdoust

**Affiliations:** a Department of Chemistry, Faculty of Science, Tabriz Branch, Islamic Azad University, PO Box 1655, Tabriz, Iran; b Department of Chemistry, University of Malaya, 50603 Kuala Lumpur, Malaysia; c Chemistry Department, Faculty of Science, King Abdulaziz University, PO Box 80203 Jeddah, Saudi Arabia; d Department of Inorganic Chemistry, Faculty of Chemistry, University of Tabriz, PO Box 5166616471, Tabriz, Iran

## Abstract

The title complex, [Cu(C_17_H_11_BrNO)_2_], lies on a centre of inversion. The chelating Schiff base anions define a square-planar N_2_O_2_ donor set. The nearly perpendicular orientation of the naphthyl residues of the chelate ring [dihedral angle = 82.12 (12)°] precludes the Cu^II^ centre from additional coordination. In the refinement, the naphthyl rings were found to be disordered over two positions.; the major component has a site occupancy of 0.667 (4).

## Related literature

For background to related Cu^II^ Schiff base compounds, see: Safaei *et al.* (2010 ▶). For a related structure, see: Dong *et al.* (2007 ▶). For specialized crystallization techniques, see: Harrowfield *et al.* (1996 ▶).
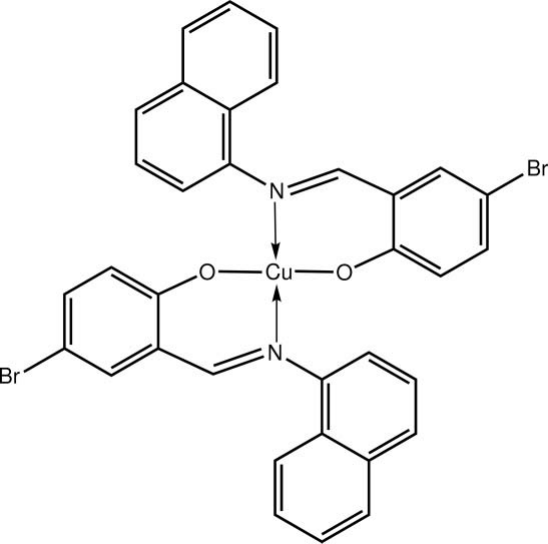


## Experimental

### 

#### Crystal data

[Cu(C_17_H_11_BrNO)_2_]
*M* *_r_* = 713.90
Monoclinic, 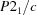

*a* = 11.4572 (6) Å
*b* = 9.5782 (3) Å
*c* = 13.8108 (6) Å
β = 114.047 (5)°
*V* = 1384.05 (10) Å^3^
*Z* = 2
Cu *K*α radiation
μ = 4.78 mm^−1^
*T* = 100 K
0.25 × 0.20 × 0.05 mm

#### Data collection

Agilent SuperNova Dual diffractometer with an Atlas detector
Absorption correction: multi-scan (*CrysAlis PRO*; Agilent, 2010 ▶) *T*_min_ = 0.381, *T*_max_ = 0.796
15204 measured reflections
2888 independent reflections
2670 reflections with *I* > 2σ(*I*)
*R*_int_ = 0.045

#### Refinement

*R*[*F* ^2^ > 2σ(*F* ^2^)] = 0.074
*wR*(*F* ^2^) = 0.157
*S* = 1.10
2888 reflections
240 parameters
69 restraints
H-atom parameters constrained
Δρ_max_ = 0.95 e Å^−3^
Δρ_min_ = −1.09 e Å^−3^

### 

Data collection: *CrysAlis PRO* (Agilent, 2010 ▶); cell refinement: *CrysAlis PRO*; data reduction: *CrysAlis PRO*; program(s) used to solve structure: *SHELXS97* (Sheldrick, 2008 ▶); program(s) used to refine structure: *SHELXL97* (Sheldrick, 2008 ▶); molecular graphics: *ORTEP-3* (Farrugia, 1997 ▶) and *DIAMOND* (Brandenburg, 2006 ▶); software used to prepare material for publication: *publCIF* (Westrip, 2010 ▶).

## Supplementary Material

Crystal structure: contains datablock(s) global, I. DOI: 10.1107/S1600536812004989/pv2510sup1.cif

Structure factors: contains datablock(s) I. DOI: 10.1107/S1600536812004989/pv2510Isup2.hkl

Additional supplementary materials:  crystallographic information; 3D view; checkCIF report

## Figures and Tables

**Fig. 1.**
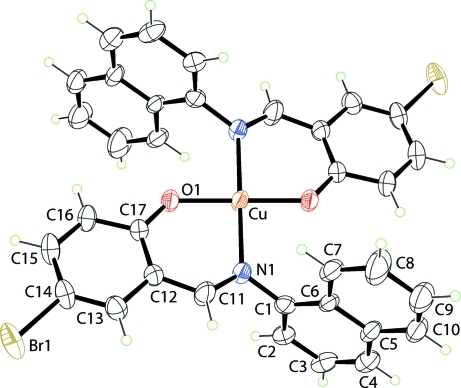
The molecular structure of the title complex showing the atom-labelling scheme and displacement ellipsoids at the 50% probability level. Only the major component of the disordered residue is shown. Symmetry code for the unlabeled atoms: -x+1, -y+1, -z+1.

**Fig. 2.**
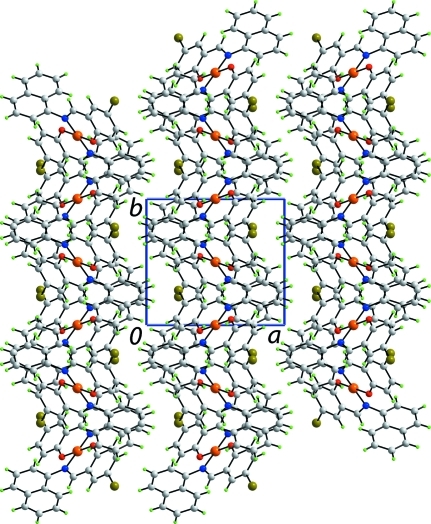
A view in projection down the c axis of the unit-cell contents of the title complex.

## supplementary crystallographic information

### Comment

There has been recent interest in the chemistry of Schiff base Cu^II^ complexes related to the title complex (Safaei *et al.*, 2010). In the title molecule (Fig. 1), the Cu^II^ atom is located on a crystallographic centre of inversion. The Cu^II^ atom is *N*,*O*-chelated by the Schiff base anions to define a square planar N_2_O_2_ geometry. The five-membered chelate ring is almost planar with a r.m.s. deviation = 0.173 °. The maximum deviation from the least-squares plane through the chelate ring is -0.178 (7) Å for the N1 atom. The naphthyl ring is almost perpendicular to the chelate ring forming a dihedral angle of 82.12 (12)°. This precludes the close approach of other atoms to the Cu^II^ centre.

The structure resembles very closely to that reported for the unsubstituted derivative (Dong *et al.*, 2007).

The disorder in the structure precludes a detailed description of the crystal packing. However, the closest interactions are of the type C—H···π involving components of the disordered naphthyl residue. Globally, molecules assemble into layers *via* weak C—H···π interactions that stack along the *a* axis (Fig. 2).

### Experimental

A solution of 1-naphthaldehyde (10 mmol) in EtOH (25 ml) was added drop-wise to a solution of 2-(aminomethyl)-4-bromophenol (10 mmol) in EtOH (15 ml). The mixture was refluxed for 9 h. The precipitate was removed by filtration and recrystallized from a MeOH solution. The ligand (0.5 mmol) was placed in one arm of a branched tube (Harrowfield *et al.*, 1996) and copper(II) perchlorate (0.5 mmol) in the other. Methanol was then added to fill both arms, the tube sealed and the ligand-containing arm immersed in a bath at 333 K, while the other was left at ambient temperature. After eight days, crystals had deposited in the arm at ambient temperature. They were filtered off, washed with acetone and ether, and air-dried. Yield: 76%. *M*.pt.: 572 K

### Refinement

Carbon-bound H-atoms were placed in calculated positions [C—H = 0.95 Å, *U*_iso_(H) = 1.2*U*_eq_(C)] and were included in the refinement in the riding model approximation.

The naphthyl ring is disordered over two positions, and each component was refined as a rigid system of 1.39 Å sides. The imino N-atom is also disordered. The C—N bond distances were restrained to within 0.01 Å of each other. The major disordered component refined to 0.667 (4); the anisotropic displacement parameters of the atoms comprising the minor component were restrained to be nearly isotropic.

### Crystal data

**Table:**

[Cu(C_17_H_11_BrNO)_2_]	*F*(000) = 710
*M**_r_* = 713.90	*D*_x_ = 1.713 Mg m^−^^3^
Monoclinic, *P*2_1_/*c*	Cu *K*α radiation, λ = 1.54184 Å
Hall symbol: -P 2ybc	Cell parameters from 6098 reflections
*a* = 11.4572 (6) Å	θ = 3.5–77.0°
*b* = 9.5782 (3) Å	µ = 4.78 mm^−^^1^
*c* = 13.8108 (6) Å	*T* = 100 K
β = 114.047 (5)°	Plate, brown
*V* = 1384.05 (10) Å^3^	0.25 × 0.20 × 0.05 mm
*Z* = 2	

### Data collection

**Table:**

Agilent SuperNova Dual diffractometer with an Atlas detector	2888 independent reflections
Radiation source: SuperNova (Cu) X-ray Source	2670 reflections with *I* > 2σ(*I*)
Mirror monochromator	*R*_int_ = 0.045
Detector resolution: 10.4041 pixels mm^-1^	θ_max_ = 77.2°, θ_min_ = 4.2°
ω scan	*h* = −14→14
Absorption correction: multi-scan (*CrysAlis PRO*; Agilent, 2010)	*k* = −12→12
*T*_min_ = 0.381, *T*_max_ = 0.796	*l* = −16→17
15204 measured reflections	

### Refinement

**Table:**

Refinement on *F*^2^	Primary atom site location: structure-invariant direct methods
Least-squares matrix: full	Secondary atom site location: difference Fourier map
*R*[*F*^2^ > 2σ(*F*^2^)] = 0.074	Hydrogen site location: inferred from neighbouring sites
*wR*(*F*^2^) = 0.157	H-atom parameters constrained
*S* = 1.10	*w* = 1/[σ^2^(*F*_o_^2^) + (0.0348*P*)^2^ + 6.8305*P*] where *P* = (*F*_o_^2^ + 2*F*_c_^2^)/3
2888 reflections	(Δ/σ)_max_ = 0.001
240 parameters	Δρ_max_ = 0.95 e Å^−^^3^
69 restraints	Δρ_min_ = −1.09 e Å^−^^3^

### Fractional atomic coordinates and isotropic or equivalent isotropic displacement parameters (Å^2^)

**Table:**

	*x*	*y*	*z*	*U*_iso_*/*U*_eq_	Occ. (<1)
Br1	0.76797 (9)	0.77617 (9)	1.04687 (5)	0.0814 (3)	
Cu	0.5000	0.5000	0.5000	0.0580 (4)	
O1	0.6132 (4)	0.4554 (4)	0.6397 (3)	0.0516 (9)	
N1	0.4092 (6)	0.6384 (7)	0.5544 (5)	0.0423 (15)	0.677 (4)
C1	0.2833 (4)	0.6955 (5)	0.4909 (3)	0.0474 (19)	0.677 (4)
C2	0.1801 (5)	0.6389 (5)	0.5059 (4)	0.048 (2)	0.677 (4)
H2	0.1940	0.5711	0.5596	0.057*	0.677 (4)
C3	0.0565 (4)	0.6817 (6)	0.4421 (5)	0.060 (4)	0.677 (4)
H3	−0.0141	0.6431	0.4523	0.071*	0.677 (4)
C4	0.0361 (3)	0.7810 (6)	0.3635 (4)	0.059 (3)	0.677 (4)
H4	−0.0484	0.8102	0.3199	0.071*	0.677 (4)
C5	0.1393 (3)	0.8375 (4)	0.3486 (3)	0.052 (2)	0.677 (4)
C6	0.2629 (3)	0.7947 (4)	0.4123 (3)	0.048 (2)	0.677 (4)
C7	0.3661 (4)	0.8513 (6)	0.3974 (5)	0.061 (3)	0.677 (4)
H7	0.4505	0.8220	0.4409	0.074*	0.677 (4)
C8	0.3457 (5)	0.9505 (7)	0.3187 (5)	0.105 (6)	0.677 (4)
H8	0.4162	0.9892	0.3085	0.126*	0.677 (4)
C9	0.2221 (6)	0.9933 (6)	0.2550 (5)	0.087 (4)	0.677 (4)
H9	0.2081	1.0612	0.2012	0.105*	0.677 (4)
C10	0.1189 (5)	0.9368 (6)	0.2699 (4)	0.067 (3)	0.677 (4)
H10	0.0344	0.9660	0.2263	0.080*	0.677 (4)
C11	0.4702 (8)	0.7009 (8)	0.6474 (5)	0.093 (3)	
H11	0.4358	0.7870	0.6578	0.112*	0.677 (4)
H11'	0.4023	0.7389	0.6619	0.112*	0.323 (4)
C12	0.5819 (5)	0.6516 (6)	0.7332 (4)	0.0497 (13)	
C13	0.6202 (6)	0.7242 (7)	0.8301 (4)	0.0550 (14)	
H13	0.5769	0.8070	0.8342	0.066*	
C14	0.7192 (6)	0.6753 (6)	0.9174 (4)	0.0558 (15)	
C15	0.7818 (6)	0.5521 (6)	0.9132 (4)	0.0587 (16)	
H15	0.8495	0.5177	0.9752	0.070*	
C16	0.7455 (5)	0.4806 (6)	0.8195 (4)	0.0494 (13)	
H16	0.7891	0.3972	0.8172	0.059*	
C17	0.6444 (5)	0.5287 (5)	0.7260 (4)	0.0430 (11)	
N1'	0.4581 (13)	0.6949 (12)	0.5452 (7)	0.037 (3)	0.323 (4)
C1'	0.3748 (7)	0.7905 (10)	0.4624 (7)	0.043 (3)	0.323 (4)
C2'	0.4313 (8)	0.8883 (12)	0.4206 (9)	0.053 (5)	0.323 (4)
H2'	0.5218	0.8950	0.4478	0.064*	0.323 (4)
C3'	0.3554 (10)	0.9763 (12)	0.3393 (10)	0.046 (5)	0.323 (4)
H3'	0.3940	1.0431	0.3108	0.056*	0.323 (4)
C4'	0.2231 (10)	0.9664 (11)	0.2996 (8)	0.069 (6)	0.323 (4)
H4'	0.1712	1.0265	0.2440	0.083*	0.323 (4)
C5'	0.1666 (7)	0.8686 (9)	0.3413 (6)	0.060 (6)	0.323 (4)
C6'	0.2424 (7)	0.7807 (7)	0.4227 (6)	0.051 (6)	0.323 (4)
C7'	0.1859 (9)	0.6829 (10)	0.4644 (9)	0.042 (4)	0.323 (4)
H7'	0.2378	0.6228	0.5200	0.050*	0.323 (4)
C8'	0.0536 (10)	0.6731 (13)	0.4248 (11)	0.082 (12)	0.323 (4)
H8'	0.0149	0.6062	0.4533	0.099*	0.323 (4)
C9'	−0.0223 (7)	0.7610 (14)	0.3434 (11)	0.063 (6)	0.323 (4)
H9'	−0.1128	0.7543	0.3163	0.075*	0.323 (4)
C10'	0.0342 (7)	0.8588 (12)	0.3017 (9)	0.067 (5)	0.323 (4)
H10'	−0.0177	0.9189	0.2461	0.080*	0.323 (4)

### Atomic displacement parameters (Å^2^)

**Table:**

	*U*^11^	*U*^22^	*U*^33^	*U*^12^	*U*^13^	*U*^23^
Br1	0.1176 (7)	0.0740 (5)	0.0377 (3)	−0.0287 (5)	0.0164 (4)	−0.0103 (3)
Cu	0.0604 (7)	0.0622 (8)	0.0352 (6)	0.0314 (6)	0.0028 (5)	−0.0068 (5)
O1	0.053 (2)	0.053 (2)	0.0371 (18)	0.0147 (18)	0.0062 (16)	−0.0025 (16)
N1	0.042 (4)	0.035 (3)	0.041 (3)	0.001 (3)	0.008 (3)	−0.003 (3)
C1	0.049 (5)	0.046 (4)	0.044 (4)	0.006 (4)	0.016 (4)	−0.014 (3)
C2	0.044 (4)	0.044 (5)	0.058 (5)	−0.001 (4)	0.023 (4)	−0.003 (4)
C3	0.037 (5)	0.052 (6)	0.083 (7)	−0.003 (4)	0.017 (5)	−0.024 (6)
C4	0.048 (6)	0.047 (5)	0.070 (6)	0.003 (4)	0.011 (5)	−0.018 (5)
C5	0.042 (5)	0.045 (4)	0.050 (5)	0.014 (4)	0.001 (4)	−0.011 (4)
C6	0.039 (4)	0.053 (6)	0.043 (5)	0.018 (4)	0.007 (4)	−0.010 (4)
C7	0.045 (5)	0.074 (7)	0.070 (6)	0.021 (5)	0.028 (5)	0.020 (5)
C8	0.100 (12)	0.104 (12)	0.104 (11)	0.032 (9)	0.035 (9)	0.057 (10)
C9	0.078 (8)	0.093 (9)	0.089 (9)	0.026 (7)	0.032 (7)	0.041 (8)
C10	0.063 (6)	0.062 (6)	0.058 (5)	0.023 (5)	0.008 (5)	0.002 (5)
C11	0.099 (6)	0.098 (6)	0.050 (4)	0.062 (5)	−0.002 (4)	−0.022 (4)
C12	0.050 (3)	0.055 (3)	0.038 (3)	0.008 (3)	0.012 (2)	−0.004 (2)
C13	0.061 (4)	0.055 (3)	0.045 (3)	−0.003 (3)	0.018 (3)	−0.006 (3)
C14	0.070 (4)	0.051 (3)	0.035 (3)	−0.017 (3)	0.010 (3)	0.000 (2)
C15	0.067 (4)	0.050 (3)	0.040 (3)	−0.016 (3)	0.003 (3)	0.009 (2)
C16	0.050 (3)	0.042 (3)	0.044 (3)	−0.006 (2)	0.006 (2)	0.009 (2)
C17	0.044 (3)	0.045 (3)	0.036 (2)	0.000 (2)	0.012 (2)	0.002 (2)
N1'	0.038 (6)	0.024 (5)	0.040 (6)	0.001 (5)	0.005 (5)	−0.006 (5)
C1'	0.052 (7)	0.043 (7)	0.034 (6)	0.010 (6)	0.018 (6)	0.002 (5)
C2'	0.062 (9)	0.049 (8)	0.054 (8)	0.013 (7)	0.029 (7)	0.007 (6)
C3'	0.053 (9)	0.040 (8)	0.053 (8)	0.013 (7)	0.029 (7)	0.005 (7)
C4'	0.083 (10)	0.073 (10)	0.057 (9)	0.015 (8)	0.036 (8)	−0.008 (7)
C5'	0.063 (9)	0.067 (10)	0.051 (9)	0.013 (8)	0.023 (7)	−0.007 (7)
C6'	0.056 (9)	0.044 (9)	0.051 (9)	0.001 (7)	0.019 (8)	0.000 (7)
C7'	0.035 (7)	0.030 (7)	0.060 (8)	−0.006 (5)	0.020 (6)	−0.002 (6)
C8'	0.075 (15)	0.083 (15)	0.084 (13)	−0.007 (9)	0.029 (9)	0.011 (9)
C9'	0.053 (9)	0.055 (9)	0.072 (9)	0.002 (7)	0.017 (7)	−0.010 (7)
C10'	0.070 (9)	0.057 (8)	0.064 (8)	0.010 (7)	0.017 (7)	−0.010 (7)

### Geometric parameters (Å, º)

**Table:**

Br1—C14	1.905 (6)	C11—H11	0.9500
Cu—O1	1.883 (3)	C11—H11'	0.9500
Cu—O1^i^	1.883 (3)	C12—C17	1.402 (7)
Cu—N1	2.010 (6)	C12—C13	1.410 (7)
Cu—N1^i^	2.010 (6)	C13—C14	1.359 (8)
Cu—N1'	2.085 (11)	C13—H13	0.9500
Cu—N1'^i^	2.085 (11)	C14—C15	1.394 (9)
O1—C17	1.302 (6)	C15—C16	1.371 (8)
N1—C11	1.328 (8)	C15—H15	0.9500
N1—C1	1.455 (6)	C16—C17	1.415 (7)
C1—C2	1.3900	C16—H16	0.9500
C1—C6	1.3900	N1'—C1'	1.472 (9)
C2—C3	1.3900	C1'—C2'	1.3900
C2—H2	0.9500	C1'—C6'	1.3900
C3—C4	1.3900	C2'—C3'	1.3900
C3—H3	0.9500	C2'—H2'	0.9500
C4—C5	1.3900	C3'—C4'	1.3900
C4—H4	0.9500	C3'—H3'	0.9500
C5—C6	1.3900	C4'—C5'	1.3900
C5—C10	1.3900	C4'—H4'	0.9500
C6—C7	1.3900	C5'—C6'	1.3900
C7—C8	1.3900	C5'—C10'	1.3900
C7—H7	0.9500	C6'—C7'	1.3900
C8—C9	1.3900	C7'—C8'	1.3900
C8—H8	0.9500	C7'—H7'	0.9500
C9—C10	1.3900	C8'—C9'	1.3900
C9—H9	0.9500	C8'—H8'	0.9500
C10—H10	0.9500	C9'—C10'	1.3900
C11—N1'	1.363 (10)	C9'—H9'	0.9500
C11—C12	1.424 (8)	C10'—H10'	0.9500
			
O1—Cu—O1^i^	180.000 (1)	N1'—C11—H11'	119.2
O1—Cu—N1	90.7 (2)	C12—C11—H11'	119.2
O1^i^—Cu—N1	89.3 (2)	C17—C12—C13	120.7 (5)
O1—Cu—N1^i^	89.3 (2)	C17—C12—C11	122.1 (5)
O1^i^—Cu—N1^i^	90.7 (2)	C13—C12—C11	116.9 (5)
N1—Cu—N1^i^	180.000 (1)	C14—C13—C12	119.8 (6)
O1—Cu—N1'	92.5 (3)	C14—C13—H13	120.1
O1^i^—Cu—N1'	87.5 (3)	C12—C13—H13	120.1
N1^i^—Cu—N1'	156.9 (4)	C13—C14—C15	120.8 (5)
O1—Cu—N1'^i^	87.5 (3)	C13—C14—Br1	118.6 (5)
O1^i^—Cu—N1'^i^	92.5 (3)	C15—C14—Br1	120.6 (4)
N1—Cu—N1'^i^	156.9 (4)	C16—C15—C14	120.0 (5)
N1'—Cu—N1'^i^	180.000 (2)	C16—C15—H15	120.0
C17—O1—Cu	129.3 (3)	C14—C15—H15	120.0
C11—N1—C1	114.5 (6)	C15—C16—C17	121.3 (6)
C11—N1—Cu	120.7 (5)	C15—C16—H16	119.4
C1—N1—Cu	124.0 (4)	C17—C16—H16	119.4
C2—C1—C6	120.0	O1—C17—C12	124.0 (5)
C2—C1—N1	117.3 (4)	O1—C17—C16	118.5 (5)
C6—C1—N1	122.5 (4)	C12—C17—C16	117.4 (5)
C3—C2—C1	120.0	C11—N1'—C1'	122.6 (9)
C3—C2—H2	120.0	C11—N1'—Cu	114.1 (7)
C1—C2—H2	120.0	C1'—N1'—Cu	118.7 (7)
C2—C3—C4	120.0	C2'—C1'—C6'	120.0
C2—C3—H3	120.0	C2'—C1'—N1'	118.5 (8)
C4—C3—H3	120.0	C6'—C1'—N1'	121.4 (8)
C5—C4—C3	120.0	C3'—C2'—C1'	120.0
C5—C4—H4	120.0	C3'—C2'—H2'	120.0
C3—C4—H4	120.0	C1'—C2'—H2'	120.0
C4—C5—C6	120.0	C2'—C3'—C4'	120.0
C4—C5—C10	120.0	C2'—C3'—H3'	120.0
C6—C5—C10	120.0	C4'—C3'—H3'	120.0
C7—C6—C5	120.0	C3'—C4'—C5'	120.0
C7—C6—C1	120.0	C3'—C4'—H4'	120.0
C5—C6—C1	120.0	C5'—C4'—H4'	120.0
C6—C7—C8	120.0	C6'—C5'—C4'	120.0
C6—C7—H7	120.0	C6'—C5'—C10'	120.0
C8—C7—H7	120.0	C4'—C5'—C10'	120.0
C9—C8—C7	120.0	C7'—C6'—C5'	120.0
C9—C8—H8	120.0	C7'—C6'—C1'	120.0
C7—C8—H8	120.0	C5'—C6'—C1'	120.0
C10—C9—C8	120.0	C6'—C7'—C8'	120.0
C10—C9—H9	120.0	C6'—C7'—H7'	120.0
C8—C9—H9	120.0	C8'—C7'—H7'	120.0
C9—C10—C5	120.0	C7'—C8'—C9'	120.0
C9—C10—H10	120.0	C7'—C8'—H8'	120.0
C5—C10—H10	120.0	C9'—C8'—H8'	120.0
N1—C11—C12	126.5 (6)	C8'—C9'—C10'	120.0
N1'—C11—C12	121.7 (7)	C8'—C9'—H9'	120.0
N1—C11—H11	116.7	C10'—C9'—H9'	120.0
N1'—C11—H11	108.6	C9'—C10'—C5'	120.0
C12—C11—H11	116.7	C9'—C10'—H10'	120.0
N1—C11—H11'	102.9	C5'—C10'—H10'	120.0
			
N1—Cu—O1—C17	−22.5 (5)	C13—C14—C15—C16	1.6 (9)
N1^i^—Cu—O1—C17	157.5 (5)	Br1—C14—C15—C16	179.9 (5)
N1'—Cu—O1—C17	0.6 (6)	C14—C15—C16—C17	−0.5 (9)
N1'^i^—Cu—O1—C17	−179.4 (6)	Cu—O1—C17—C12	10.2 (8)
O1—Cu—N1—C11	27.1 (7)	Cu—O1—C17—C16	−171.3 (4)
O1^i^—Cu—N1—C11	−152.9 (7)	C13—C12—C17—O1	179.1 (6)
N1'—Cu—N1—C11	−67.6 (7)	C11—C12—C17—O1	5.6 (10)
N1'^i^—Cu—N1—C11	112.4 (7)	C13—C12—C17—C16	0.6 (8)
O1—Cu—N1—C1	−163.7 (6)	C11—C12—C17—C16	−172.9 (7)
O1^i^—Cu—N1—C1	16.3 (6)	C15—C16—C17—O1	−179.2 (5)
N1'—Cu—N1—C1	101.7 (10)	C15—C16—C17—C12	−0.6 (8)
N1'^i^—Cu—N1—C1	−78.3 (10)	N1—C11—N1'—C1'	95.6 (15)
C11—N1—C1—C2	−89.0 (7)	C12—C11—N1'—C1'	−154.6 (10)
Cu—N1—C1—C2	101.2 (5)	N1—C11—N1'—Cu	−60.0 (8)
C11—N1—C1—C6	96.0 (8)	C12—C11—N1'—Cu	49.8 (13)
Cu—N1—C1—C6	−73.9 (6)	O1—Cu—N1'—C11	−27.8 (9)
C6—C1—C2—C3	0.0	O1^i^—Cu—N1'—C11	152.2 (9)
N1—C1—C2—C3	−175.2 (5)	N1—Cu—N1'—C11	58.1 (7)
C1—C2—C3—C4	0.0	N1^i^—Cu—N1'—C11	−121.9 (7)
C2—C3—C4—C5	0.0	O1—Cu—N1'—C1'	175.5 (9)
C3—C4—C5—C6	0.0	O1^i^—Cu—N1'—C1'	−4.5 (9)
C3—C4—C5—C10	180.0	N1—Cu—N1'—C1'	−98.5 (14)
C4—C5—C6—C7	180.0	N1^i^—Cu—N1'—C1'	81.5 (14)
C10—C5—C6—C7	0.0	C11—N1'—C1'—C2'	111.9 (12)
C4—C5—C6—C1	0.0	Cu—N1'—C1'—C2'	−93.5 (9)
C10—C5—C6—C1	180.0	C11—N1'—C1'—C6'	−70.8 (15)
C2—C1—C6—C7	180.0	Cu—N1'—C1'—C6'	83.8 (10)
N1—C1—C6—C7	−5.0 (5)	C6'—C1'—C2'—C3'	0.0
C2—C1—C6—C5	0.0	N1'—C1'—C2'—C3'	177.3 (9)
N1—C1—C6—C5	175.0 (5)	C1'—C2'—C3'—C4'	0.0
C5—C6—C7—C8	0.0	C2'—C3'—C4'—C5'	0.0
C1—C6—C7—C8	180.0	C3'—C4'—C5'—C6'	0.0
C6—C7—C8—C9	0.0	C3'—C4'—C5'—C10'	180.0
C7—C8—C9—C10	0.0	C4'—C5'—C6'—C7'	180.0
C8—C9—C10—C5	0.0	C10'—C5'—C6'—C7'	0.0
C4—C5—C10—C9	180.0	C4'—C5'—C6'—C1'	0.0
C6—C5—C10—C9	0.0	C10'—C5'—C6'—C1'	180.0
C1—N1—C11—N1'	−97.8 (10)	C2'—C1'—C6'—C7'	180.0
Cu—N1—C11—N1'	72.5 (9)	N1'—C1'—C6'—C7'	2.8 (9)
C1—N1—C11—C12	167.1 (8)	C2'—C1'—C6'—C5'	0.0
Cu—N1—C11—C12	−22.6 (13)	N1'—C1'—C6'—C5'	−177.2 (9)
N1—C11—C12—C17	2.5 (14)	C5'—C6'—C7'—C8'	0.0
N1'—C11—C12—C17	−40.5 (13)	C1'—C6'—C7'—C8'	180.0
N1—C11—C12—C13	−171.2 (9)	C6'—C7'—C8'—C9'	0.0
N1'—C11—C12—C13	145.8 (9)	C7'—C8'—C9'—C10'	0.0
C17—C12—C13—C14	0.5 (9)	C8'—C9'—C10'—C5'	0.0
C11—C12—C13—C14	174.3 (7)	C6'—C5'—C10'—C9'	0.0
C12—C13—C14—C15	−1.6 (9)	C4'—C5'—C10'—C9'	180.0
C12—C13—C14—Br1	−179.9 (5)		

Symmetry code: (i) −*x*+1, −*y*+1, −*z*+1.
